# Supplementary material for: Forecast of myocardial infarction incidence, events and prevalence in England to 2035 using a microsimulation model with endogenous disease outcomes
Source: PLoS One. 2022 Jun 30;17(6):e0270189. doi: 10.1371/journal.pone.0270189 (PMC9246106; doi:10.1371/journal.pone.0270189)

**Forecast of myocardial infarction incidence, events and prevalence in England to 2035 using a microsimulation model with endogenous disease outcomes**

Supporting information

Peter Scarborough, Asha Kaur and Linda Cobiac

Detailed description of the microPRIME model

A schematic showing the linked R modules and data repositories that provide the structure of the microPRIME model are shown in figure S1. A description of each of the R modules is provided below. Each of the R modules is available to download at <https://github.com/PeteScarbs/microPRIME/>.

**Figure S1:** Schematic of the microPRIME model


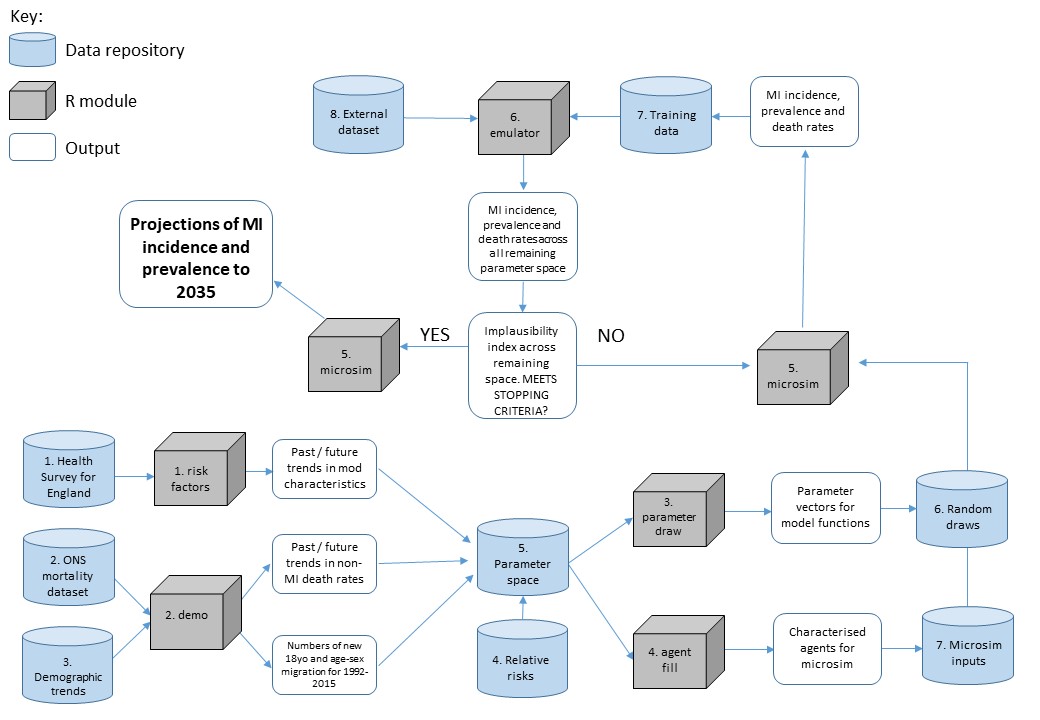


***risk factors:*** This R module estimates the joint distribution in the English population of the continuous variables age, BMI, systolic blood pressure (SBP), total cholesterol, and binary variables current smoking, doctor-diagnosed diabetes, and previous myocardial infarction (MI). It also estimates trends by age and year in each of these variables between 1993 and 2014 (all analyses are stratified by sex).

All of the data for this module are drawn from the Health Survey for England (HSE) series between 1993 and 2014. The HSE is a representative sample of approximately 8,000 adults per year. Data are collected by self-completed questionnaire for age, sex, current smoking, diabetes and previous MI. A trained nurse also measures height and weight for estimates of BMI, as well as SBP. A blood sample is taken by the nurse which is analysed to estimate total cholesterol levels (NatCen, 2015). Age, sex, BMI and smoking data are collected annually, and data on the remaining variables is collected in years where the survey had a particular focus on cardiovascular disease and risk factors (1998, 2003, 2005, 2006, 2011).

For each of the surveys we identified the variables of interest (when they were available) and compiled all the data into a single dataset. We restricted the dataset to over 18s only. Over this time period, the HSE had boost samples of older populations, children and ethnic minorities. As our dataset was restricted to adults, the boost samples for children were not included. We did not include the boost samples for ethnic minorities as doing so would affect the representativeness of the sample to the general population. We did, however, include the boost sample of the older populations. This is because all of our analyses were adjusted by age, so additional data points in older age groups will not affect the representativeness of the sample.

For BMI, SBP and total cholesterol we assessed the shape of the distribution within the male and female populations by observing Cullen and Frey graphs and by comparing the goodness-of-fit of observed data with normal, lognormal, gamma and Weibull distributions. We selected the distribution which demonstrated the lowest Kolgorov-Smirnov statistic. This resulted in lognormal distributions for BMI and SBP and gamma distributions for total cholesterol.

For a given year, representing the start of the simulation process (for this paper all simulations began in the baseline year of 1998), we extracted data on the joint distribution of age, BMI, SBP, total cholesterol, smoking and diabetes using Pearson’s r coefficient for all two-way correlations. For this process we used a complete case analysis on all individuals from any of the three survey years centred on the baseline year.

To estimate trends in risk factors we used a different approach for BMI than for the other risk factors. For BMI, we used functions that describe how both mean BMI and the standard deviation of BMI varies over time (therefore allowing the distribution of BMI in the population to change over time). We fit two sets of functions. The first set were derived using linear regression models with a quadratic function for calendar year, which result in projected BMI curves that peak around 2020 and begin to fall thereafter. The second set were derived using non-linear models with a restraint that (all else being equal) BMI cannot reduce over time. These models result in projections that head towards an asymptote slightly higher than the 2020 peak from the linear models. Both sets of models have very high agreement over the period were they are fitted to the data, and only diverge in the projections. A full description of how these models were built and fit to the data is provided in Cobiac and Scarborough (2021). In this forecast paper we used the non-linear models that project mean BMI that approaches an asymptote after 2020.

We used logistic regression models to estimate trends in odds of current smoking and doctor-diagnosed diabetes and linear regression models to estimate trends in SBP and total cholesterol. All models were stratified by sex. For all outcomes, we built models with exposure variables age and calendar year, plus quadratic terms for both and interaction terms. We used likelihood ratio tests to assess whether model fit improved by adding quadratic and interaction terms, with p = 0.05 as a threshold for decision making. Full details of the risk factor trend models are provided in table S1. Modelled trends over the period 1993 to 2035 are shown in Figure S2.

**Table S1:** Details of the models used to project risk factor trends over time.

| *Risk factor* | *Fitted model for trend* |
| --- | --- |
| BMI | Mean BMI ~ sex + age + age^2^ + sex*age + sex*age^2^ – a*exp(-b*year)  SD BMI ~ meanBMI + sex + age + age^2^ + sex*age + sex*age^2^ – a*exp(-b*year) |
| SBP | Men: SBP ~ age + year + age^2^ + year^2^  Women: SBP ~ age + year + age^2^ + year^2^ |
| Total cholesterol | Men: Cholesterol ~ age + year + age^2^  Women: Cholesterol ~ age + year + age^2^ + year^2^ |
| Smoking | Men: logit(smoking) ~ age + year + age^2^  Women: logit(smoking) ~ age + year + age^2^ + year^2^ |
| Diabetes | Men: logit(diabetes) ~ age + year + age^2^ + year^2^  Women: logit(diabetes) ~ age + year + age^2^ |

To demonstrate how well the models correspond with the external dataset, we calculated the median and 95% uncertainty intervals for estimates of mean (or prevalence) of each risk factor in 2011 across the 450 model runs. These are shown in Table S2 and compared with external estimates from the 2011 Health Survey for England. There are 40 risk factor-age-sex groups (5 risk factors x 4 age categories x 2 sexes). For 34 of these groups, the 95% uncertainty intervals from the model runs overlap with the 95% confidence intervals from the Health Survey for England.

**Figure S2:** Modelled trends in risk factors, 1993-2035


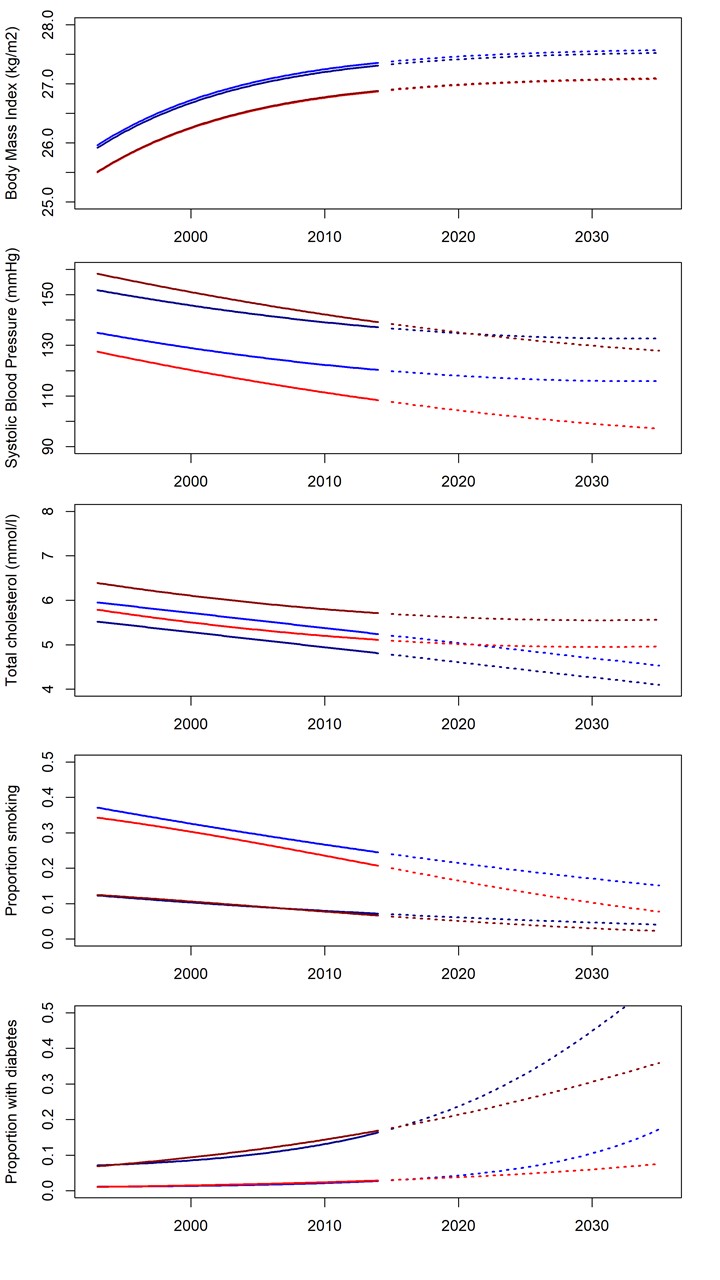


*Hard lines are trends based on regression models fit to data from the Health Survey for England data between 1993 and 2014. Dotted lines are projections of the trends to 2035. All lines represent average pathways – the microPRIME model allows for variation around these lines for different agents. Blue = Men aged 40; Dark blue = Men aged 80; Red = Women aged 40; Dark Red = Women aged 80*

**Table S2:** Comparison of risk factor estimates for 2011 produced by the microPRIME model and measured in the 2011 Health Survey for England

|  | **MEN** | | **WOMEN** | |
| --- | --- | --- | --- | --- |
| *Age group* | *Modelled estimates (95% UI)* | *Measured estimates (95% CI)* | *Modelled estimates (95% UI)* | *Measured estimates (95% CI)* |
| Mean BMI (kg/m^2^) | | | | |
| 55-64 | 28.0 (27.5 - 28.5) | 28.8 (28.4 - 29.2) | 27.5 (27.1 – 28.0) | 28.1 (27.7 - 28.5) |
| 65-74 | 27.5 (27.0 - 28.0) | 28.5 (28.1 - 28.9) | 27.2 (26.8 - 27.7) | 28.2 (27.8 - 28.7) |
| 75-84 | 27.2 (26.7 - 27.7) | 28.5 (28.0 - 29.0) | 26.8 (26.3 - 27.4) | 28.5 (27.9 - 29.1) |
| 85+ | 26.6 (26.2 - 27.2) | 26.5 (25.5 - 27.5) | 26.5 (26.0 - 27.0) | 26.2 (25.1 - 27.3) |
| Mean systolic blood pressure (mmHg) | | | | |
| 55-64 | 135.5 (132.7 - 138.2) | 133.0 (131.5 - 134.6) | 132.3 (130.1 - 135.2) | 130.1 (128.5 - 131.7) |
| 65-74 | 136.6 (133.9 - 139.3) | 133.6 (131.8 - 135.4) | 134.2 (132.0 - 137.1) | 135.2 (133.4 - 137.1) |
| 75-84 | 137.2 (134.6 - 140.0) | 135.6 (133.0 - 138.1) | 136.4 (134.1 - 139.2) | 136.6 (134.1 - 139.1) |
| 85+ | 138.8 (136.0 - 141.5) | 133.1 (127.5 - 138.7) | 139.7 (137.4 - 142.8) | 135.2 (130.0 - 140.3) |
| Mean total cholesterol (mmol/l) | | | | |
| 55-64 | 5.1 (5.1 - 5.2) | 5.4 (5.2 - 5.5) | 5.5 (5.4 - 5.6) | 6.0 (5.8 - 6.1) |
| 65-74 | 5.0 (4.9 - 5.0) | 5.0 (4.8 - 5.1) | 5.5 (5.4 - 5.6) | 5.7 (5.6 - 5.8) |
| 75-84 | 4.8 (4.7 - 4.9) | 4.7 (4.5 - 4.9) | 5.6 (5.5 - 5.7) | 5.5 (5.3 - 5.6) |
| 85+ | 4.7 (4.6 - 4.8) | 4.6 (4.2 - 5.0) | 5.7 (5.6 - 5.8) | 5.3 (4.9 - 5.6) |
| Proportion who currently smoke | | | | |
| 55-64 | 0.16 (0.12 - 0.22) | 0.20 (0.17 - 0.23) | 0.15 (0.11 - 0.21) | 0.18 (0.15 - 0.21) |
| 65-74 | 0.10 (0.07 - 0.14) | 0.11 (0.09 - 0.14) | 0.10 (0.06 - 0.15) | 0.15 (0.12 - 0.17) |
| 75-84 | 0.05 (0.03 - 0.08) | 0.06 (0.03 - 0.08) | 0.05 (0.03 - 0.09) | 0.04 (0.02 - 0.06) |
| 85+ | 0.02 (0.01 - 0.04) | 0.02 (0.00 - 0.05) | 0.03 (0.01 - 0.05) | 0.04 (0.01 - 0.07) |
| Proportion with doctor-diagnosed diabetes | | | | |
| 55-64 | 0.08 (0.02 - 0.36) | 0.11 (0.09 - 0.14) | 0.05 (0.02 - 0.22) | 0.06 (0.05 - 0.08) |
| 65-74 | 0.12 (0.02 - 0.51) | 0.16 (0.13 - 0.19) | 0.08 (0.03 - 0.36) | 0.10 (0.07 - 0.12) |
| 75-84 | 0.15 (0.02 - 0.61) | 0.19 (0.14 - 0.23) | 0.09 (0.04 - 0.48) | 0.15 (0.11 - 0.18) |
| 85+ | 0.14 (0.02 - 0.66) | 0.25 (0.16 - 0.34) | 0.10 (0.03 - 0.58) | 0.12 (0.06 - 0.17) |

*UI – Uncertainty Intervals; CI – Confidence Intervals; Green filled cells indicate that uncertainty and confidence intervals overlap; Red cells indicate that uncertainty and confidence intervals do not overlap.*

***demo:*** This R module generates age and sex specific mortality rates for ‘other mortality’ (i.e. any deaths not associated with MI) for any year. We combined data on population estimates by sex and single year of age with number of deaths from all causes and from MI (any deaths where MI is described as main or contributing factor on the death certificate) by sex and five year age group. Data were provided by the Office for National Statistics for every year between 1992 and 2015 (inclusive). We estimated the non-MI mortality rate by subtracting MI mortality from all mortality and dividing by the number of people in each sex and five year age group. We smoothed the mortality data to provide estimates for single year of age by assuming exponential growth in mortality rates by age. We then fitted sex-specific models across all data points with the log-transformed non-MI mortality rate as the dependent variable and age and year as the independent variables. The model parameters were used to estimate age, sex and year-specific non-mortality rates in the *microsim* module.

***agent fill:*** This R module generates the population of agents that are used in the *microsim* module, based on data generated in the *demo* and *risk factor* modules. The module can generate any number of agents. For the purpose of this paper, we generated 114,000 (this number was a trade-off between allowing high enough numbers of events to reduce stochastic uncertainty in microsimulation results and keeping model computation time manageable). Using the data on joint distributions generated by the *risk factor* module, we used a copula to generate two matrices of order n/2 x 6 (where n is the number of agents – halved as we build separate matrices for men and women – and 6 columns for age, BMI, SBP, total cholesterol, smoking and diabetes). Each of the columns in these matrices are continuous variables in the range [0,1] and have the property that they retain the same marginal correlations between any two columns as was observed in the joint distribution of age and the risk factors generated by *risk factor*. We transformed the first four columns into age, BMI, SBP and total cholesterol such that they follow the age distribution seen in the English population in the baseline year and the distribution of the continuous risk factors observed in the HSE data. For smoking and diabetes, the data were left as the continuous variables with range [0,1] – these are transformed into binary variables of current smoking and diagnosed diabetes in the *microsim* module. We then added another variable to represent whether the agent has had a previous MI. We used HSE data to run logistic regression models for men and women separately with previous MI as dependent and age (with a quadratic term) as the independent variables. These model results were used to attach probability of having previous MI to the matrices. We then ran stochastic tests across the matrices to convert the probability into a binary variable where 1 represents a previous MI. The two matrices (for men and women) were combined into a single dataset of 114,000 agents for use in the *microsim* module.

***parameter draw:*** The goal of this module is to draw random sets of model parameters that vary between each iteration of the *microsim* module. Outcomes of the *microsim* module (MI incidence, event and prevalence rates by sex, age group and calendar year) vary between iterations for two reasons: *stochastic* variation, which results from the probabilistic nature of resolving state transitions for agents (see *microsim*); and *deterministic* variation, which results from differences in the functions that determine state transitions within each iteration. These deterministic differences are due to the functions in each iteration which depend upon the set of parameters that are drawn by the *parameter draw* module. The parameters that vary between each iteration are shown below, with the range over which the parameters are drawn randomly with a uniform distribution. Our aim was to initially draw from a very broad parameter space, so where possible the range of possible parameter estimates was taken from 99% confidence intervals around the best estimate, which we sometimes derived from published 95% confidence intervals.

**Table S3:** Model parameters that vary between iterations

| *Parameter name* | *Description* | *Range* |
| --- | --- | --- |
| alpha.30dcf.m | Intercept parameter for 30 day case fatality from MI by age, men | 99% confidence intervals from model of log(30 day case fatality) ~ age, fit against data from Smolina (2012) |
| alpha.30dcf.f | Intercept parameter for 30 day case fatality from MI by age, women | 99% confidence intervals from model of log(30 day case fatality) ~ age, fit against data from Smolina (2012) |
| treatment.cf.mi.m | Annual % reduction in case fatality, men | 3.5% - 6.5%, based on data from Smolina (2012) |
| treatment.cf.mi.f | Annual % reduction in case fatality, women | 3.5% - 6.5%, based on data from Smolina (2012) |
| bmi.m.r1525 | Relative risk for 5kg/m2 increase in BMI in range 15-25, men | 99% confidence intervals from Prospective Studies Collaboration (2009) |
| bmi.m.r2550 | Relative risk for 5kg/m2 increase in BMI in range 25-50, men | 99% confidence intervals from Prospective Studies Collaboration (2009) |
| bmi.f.r1525 | Relative risk for 5kg/m2 increase in BMI in range 15-25, women | 99% confidence intervals from Prospective Studies Collaboration (2009) |
| bmi.f.r2550 | Relative risk for 5kg/m2 increase in BMI in range 25-50, women | 99% confidence intervals from Prospective Studies Collaboration (2009) |
| sbp.alpha.m | Intercept parameter for linear change in RR by age for 20mmHg reduction in SBP, men | 99% confidence intervals from model of RR ~ age, fit against data from Prospective Studies Collaboration (2002) |
| sbp.alpha.f | Intercept parameter for linear change in RR by age for 20mmHg reduction in SBP, women | 99% confidence intervals from model of RR ~ age, fit against data from Prospective Studies Collaboration (2002) |
| tcho.alpha.m | Intercept parameter for sigmoid function relating RR for 1mmol/l reduction in cholesterol with age, men | 99% confidence intervals from model of (1+exp(-RR))^-1^ ~ age, fit against data from Prospective Studies Collaboration (2007) |
| tcho.alpha.f | Intercept parameter for sigmoid function relating RR for 1mmol/l reduction in cholesterol with age, women | 99% confidence intervals from model of (1+exp(-RR))^-1^ ~ age, fit against data from Prospective Studies Collaboration (2007) |
| smok.alpha.m | Intercept parameter for linear change in RR by age for smoking, men | 99% confidence intervals from model of RR ~ age, fit against data from Thun (2000) |
| smok.alpha.f | Intercept parameter for linear change in RR by age for smoking, women | 99% confidence intervals from model of RR ~ age, fit against data from Thun (2000) |
| diab.m | Relative risk for diabetes, men | 99% confidence intervals from Peters (2014) |
| diab.f | Relative risk for diabetes, women | 99% confidence intervals from Peters (2014) |
| bmi.trend.age.m | Parameter linking change in BMI by age, men | 99% confidence intervals from trend models described in Cobiac and Scarborough (2021) |
| bmi.trend.year.m | Parameter linking change in BMI by year, men | 99% confidence intervals from trend models described in Cobiac and Scarborough (2021) |
| bmi.trend.age.f | Parameter linking change in BMI by age, women | 99% confidence intervals from trend models described in Cobiac and Scarborough (2021) |
| bmi.trend.year.f | Parameter linking change in BMI by year, women | 99% confidence intervals from trend models described in Cobiac and Scarborough (2021) |
| sbp.trend.age.m | Parameter linking change in SBP by age, men | 99% confidence intervals from bespoke trend models described under *risk factors* module. |
| sbp.trend.age.f | Parameter linking change in SBP by age, women | 99% confidence intervals from bespoke trend models described under *risk factors* module. |
| sbp.trend.year.m | Parameter linking change in SBP by year, men | 99% confidence intervals from bespoke trend models described under *risk factors* module. |
| sbp.trend.year.f | Parameter linking change in SBP by year, women | 99% confidence intervals from bespoke trend models described under *risk factors* module. |
| tcho.trend.age.m | Parameter linking change in cholesterol by age, men | 99% confidence intervals from bespoke trend models described under *risk factors* module. |
| tcho.trend.age.f | Parameter linking change in cholesterol by age, women | 99% confidence intervals from bespoke trend models described under *risk factors* module. |
| tcho.trend.year.m | Parameter linking change in cholesterol by year, men | 99% confidence intervals from bespoke trend models described under *risk factors* module. |
| tcho.trend.year.f | Parameter linking change in cholesterol by year, women | 99% confidence intervals from bespoke trend models described under *risk factors* module. |
| smok.trend.age.m | Parameter linking change in smoking by age, men | 99% confidence intervals from bespoke trend models described under *risk factors* module. |
| smok.trend.age.f | Parameter linking change in smoking by age, women | 99% confidence intervals from bespoke trend models described under *risk factors* module. |
| smok.trend.year.m | Parameter linking change in smoking by year, men | 99% confidence intervals from bespoke trend models described under *risk factors* module. |
| smok.trend.year.f | Parameter linking change in smoking by year, women | 99% confidence intervals from bespoke trend models described under *risk factors* module. |
| diab.trend.age.m | Parameter linking change in diabetes by age, men | 99% confidence intervals from bespoke trend models described under *risk factors* module. |
| diab.trend.age.f | Parameter linking change in diabetes by age, women | 99% confidence intervals from bespoke trend models described under *risk factors* module. |
| diab.trend.year.m | Parameter linking change in diabetes by year, men | 99% confidence intervals from bespoke trend models described under *risk factors* module. |
| diab.trend.year.f | Parameter linking change in diabetes by year, women | 99% confidence intervals from bespoke trend models described under *risk factors* module. |
| prev.alpha.m | Intercept parameter for change in RR of MI event by age and time since previous MI, men | 99% confidence intervals from model of log(RR) ~ age + time since MI, fit against data from Smolina (2012) |
| prev.alpha.f | Intercept parameter for change in RR of MI event by age and time since previous MI, women | 99% confidence intervals from model of log(RR) ~ age + time since MI, fit against data from Smolina (2012) |

The *parameter draw* module uses Latin Hypercube Sampling (LHS) to generate a sample of m vectors each of length n, where m is the number of iterations to be modelled, and n is the number of model parameters. For the analyses reported here, each model run had 450 iterations (see *emulator* for justification of number of iterations) and there were 38 model parameters. The LHS method ensures that there is equal density of coverage across the [0,1]^n^ vector space that is being sampled. After generation of the sample, each parameter is converted from [0,1] to the range described in table S3.

***microsim:*** The *microsim* module draws on data generated by each of the previous modules and uses them to simulate individuals (agents) progressing through time. Each agent progresses through multiple time steps, each representing a year. The agents are entered into the model with data on gender, age, BMI, SBP, total cholesterol, a binary variable stating whether or not they have suffered a previous MI and two continuous variables between [0,1] representing probability of smoking and diabetes (see *agent fill* module). After each year, they age and BMI, SBP and total cholesterol progress following trend functions described above. The smoking and diabetes variables are converted into binary variables describing presence or absence by comparison with age, sex and year-specific estimates of the prevalence of smoking and diabetes, which are generated using trend functions described above – if the continuous variable is above (1 – prevalence) then the binary variable is 1 (i.e. current smoker / has doctor-diagnosed diabetes), and is 0 otherwise.

Three events can happen to an agent at any time point: 1. they can have an MI, 2. they can die from an MI, 3. they can die of an unrelated cause. It is not possible for an agent to die from an MI without first having an MI, although both of those events can happen in a single time step. The state transition probabilities dictating these events are 1. the incidence rate of MI, 2. the death rate from MI (NB: this incorporates both short term case-fatality and long-term increased mortality risk), 3. the death rate from unrelated causes. The unique selling point of the microPRIME model is that population-level incidence, prevalence and death rates for MI emerge from the model (i.e. they are endogenous variables). To enable this, the microPRIME model generates an age- and sex-specific *static* incidence rate for MI – this rate controls the incidence of MI for the lowest risk group (with BMI 15kg/m^2^ or less, SBP 90mmHg or less, total cholesterol 2mmol/l or less, non-smoker, and no previous diagnosis of diabetes or MI) for each sex and for any age. As the name implies, this static rate does not change over time. All changes in population-level incidence, prevalence and death rates in microPRIME are because of changes over time in the risk factors and the effect of treatments within the population, which operate on this static incidence rate through the parameters shown in table S2.

Each iteration of the microPRIME model generates new static incidence rates. These are calibrated using age and sex-specific incidence rates of MI for 2007 from a linked dataset of all hospital episodes and death certificates in England (Smolina, 2012), and a dataset of 100,000 agents generated using the *agent fill* module based on HSE data from 2006 (NB: this year was chosen as it was the closest to 2007 from which a complete set of risk factor data were available from the HSE data). The functions to estimate relative risks for MI are applied to all of the agents in the calibration dataset, and the median of the multiplicatively combined relative risks for each sex and single year of age are calculated. The MI incidence rates for 2007 are divided by the median relative risks to generate the static incidence rates. For each iteration the incidence rates for 2007 remain the same, but the relative risks are different as the functions to generate them have randomly assigned parameters – therefore the static incidence rates change for every iteration. An example of this process is shown in figure S3.

Each alive agent in the model is at risk of a new MI with their probability of a new MI event based on their age and sex, risk factor status, whether they have had a previous MI and the effect of treatment. An agent can have up to 4 MI events in the model, but the fourth event is always fatal. For all other MI events, the age and sex-specific risk of death from MI within 30 days is estimated based on the linked dataset of hospital episodes and death certificates (Smolina, 2012) adjusted for treatment effects. Agents that survive have an increased risk of another MI event, which is estimated as another relative risk (combined multiplicatively with relative risks for other risk factors), and is a function of age at MI, sex and time since the last MI event. This function is parameterised using long-term survival curves for incident MI cases (Smolina, 2012). The additional risk of further MI conveys additional risk of death for all agents who have had an incident case.

The remaining transition factor – risk of death from unrelated cause – is an exogenous variable parameterised using English trend data (1992 - 2012) on all-cause mortality minus mortality where MI is mentioned on the death certificate (see *demo*).

The *microsim* module has two purposes – to generate training data for the *emulator* module, and to run the final model with projected incidence and prevalence rates to 2035 once the emulation process has completed. For both these purposes, *microsim* must collect output data. For the training data, it is necessary to collect data on model-generated incidence, prevalence and event rates that coincide with the external datasets that are being used for the process (see *emulator*). The *microsim* module can be set to collect data from any period and for any age group, for the purpose of this paper it collects data for each sex for four age groups (55-64; 65-74; 75-84; 85+) and for seven years (1999, 2003, 2005, 2006, 2007, 2011, 2018). It also estimates the statistical variance around these rates, based on the number of agents included in each calculation. For the projection of trends in the final model run, the same age and sex groups are used, but incidence, prevalence and event rates are estimated for every fourth year between 1999 and 2035.

**Figure S3:** Example of how the static incidence rates are generated. (A) Incidence rate for first MI, from Smolina et al. (2012); (B) Median relative risk (RR) for MI by age and sex (multiplicative combination of RR for BMI, SBP, cholesterol, smoking and diabetes), using best estimates for RR functions and HSE2006 data for the population; (C) Static incidence rates for first MI, generated by dividing incidence rates in (A) by median RRs in (B). *For (A) and (B), dots give point estimates and lines show a best fit to these data using a cubic spline with knots at the points marked by dashed lines. Results for men are in blue, and for women are in red.*


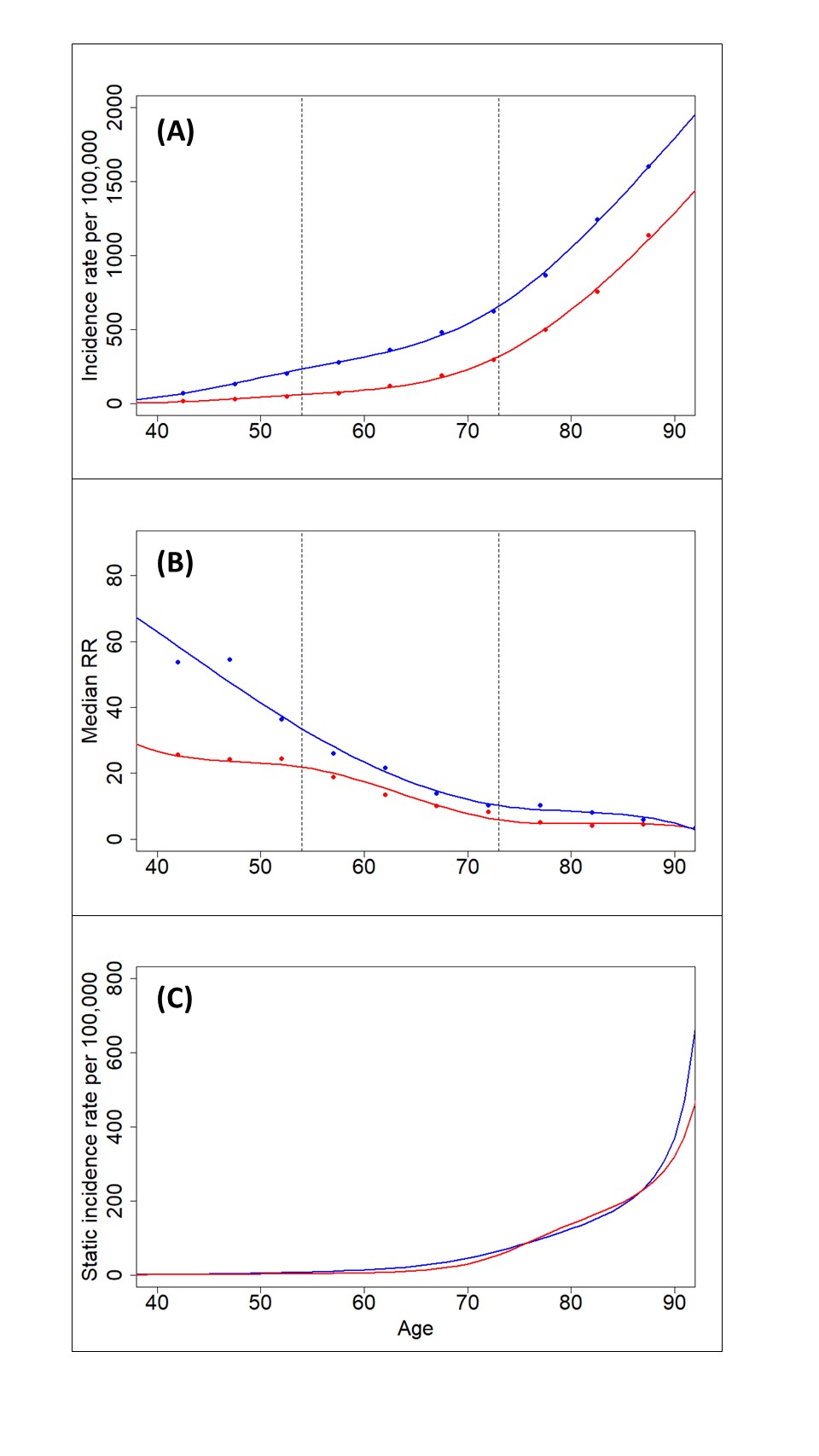


***emulator:*** The purpose of the emulator is to reduce the uncertainty range around microPRIME estimates of incidence, event and prevalence rates for MI, by identifying sets of parameters that produce implausible model outcomes based on comparison with external datasets. As shown in figure S1, the *microsim* and *emulator* modules operate in a loop that is only broken when pre-determined stopping criteria are achieved. This loop proceeds as follows. The *microsim* module generates model outcomes over a number of iterations (for the analyses in this paper, there are 450 iterations per model run) and each iteration is based on a unique vector of model parameters which control how the model operates (see *parameter draw*). The model outcomes and their associated parameter vectors are saved as training data for the *emulator* module, which uses them to estimate model outcomes for a much larger set of parameter vectors (for this paper, *emulator* was run over 2,000,000 parameter vectors). The emulated model outcomes are compared with estimates of incidence, prevalence and event rates for MI from external datasets, and those that are too distant from the external estimates are deemed implausible and removed from the set of available parameter vectors. A new random sample of 450 parameter vectors are drawn from the remaining set, and these are used in another run of the *microsim* module, which in turn generates new training data for the *emulator* module. Since each successive run of the *microsim* module is based on parameters that are producing more plausible model outcomes, the *emulator* module can generate closer fitting estimated outcomes, and hence find more implausible parameter vectors to exclude. When the stopping criteria are achieved, a final set of 450 parameter vectors are sampled from the remaining vector space, and these parameters are used for the *microsim* model run that projects estimates to 2035.

The *emulator* module initially draws a set of 2,000,000 parameter vectors using Latin Hypercube Sampling (see *parameter draw*). It then builds an emulator for each of the model outcomes using the ‘emulator’ package in RStudio (Hankin, 2015). Limited to a maximum of 100 iterations, scales for each of the emulators are generated from the training data (Oakley, 2004). The emulators are then applied to the 450 parameter vectors from the training data, and correlations between the model-estimated outcomes (from the training data) and the emulated model outcomes are obtained. For all emulators where this correlation, measured by Pearson’s r, is greater than 0.6, the emulator is then run across the entire of the remaining parameter vectors.

For each of the model outcomes that are being emulated, one iteration of the microPRIME model provides one data point used to fit the emulators. For our analyses we used 450 iterations, so each emulator was fit using 450 data points. As a rule of thumb, Andrianakis et al. (2015) suggest there should be at least 10 data points for every parameter that is allowed to vary in the model. For our analyses we have 38 varying parameters, (19 for men and 19 for women). Since separate emulators are built for men and women, this means that we have over 23 data points per varying parameter. There are two methods to improve the fit of the emulators. First, you can increase the number of iterations in the training data – this provides a greater number of data points to fit the emulators. Second, you can increase the number of agents in each iteration – this reduces stochastic variation in model outcomes, which in turn reduces the random noise in the emulator fitting process. Both of these methods increase the time taken for both the microsim and emulator modules to run, so selecting appropriate numbers of agents and iterations requires a balance. We tested the microPRIME model over a range of 50,000-114,000 agents and 380-900 iterations, to observe the computation time and the number of emulated outcomes for which correlation with modelled outcomes achieved a minimum threshold (r > 0.6). We found that increasing the number of agents had a bigger impact on emulator fit than increasing the number of iterations. Therefore, we prioritised a high number of agents and moderate number of iterations in our final model.

All of the emulated model outcomes are then compared with estimates from external datasets, which are itemised in table S4.

**Table S4:** External datasets used by the *emulator* module

| Model outcome | Years available in external dataset | External dataset |
| --- | --- | --- |
| Incidence of first MI per 100,00 | 2007 | Linked hospital episodes and death certificates (Smolina, 2012) |
| Prevalence of having had previous MI | 2003, 2005, 2006, 2011 | Health Survey for England (NatCen, 2016) |
| Age-standardised MI event rate per 100,000 | 1999, 2003, 2007 | Linked hospital episodes and death certificates (Smolina, 2012) |

*Note that all of the incidence and prevalence outcomes in the external datasets are available for each sex and for four age groups (55-64; 65-74; 75-84; 85+) with the exception of prevalence estimates in 2005, where estimates for 55-64 year olds are not available.*

To compare the emulated outcomes with the estimates from external datasets an implausibility function is used. This is based on a similar function developed by Andrianakis et al. (2016) and is defined as follows:

$$T\left( \boldsymbol{x} \right)= \frac{\left| z-g\left( \boldsymbol{x} \right) \right|}{\sqrt{var\left( c \right)+var\left( e \right)+var\left( o \right))}}$$

Where:

- z is the measure of the outcome from the external dataset
- g(**x**) is the emulated estimate of the outcome for parameter vector **x**
- var(c) is the variance in the emulated set of outcomes
- var(e) is the variance in the training data set of outcomes
- var(o) is the variance in the measured outcomes from the external dataset

For each *emulator* run, the next selection of parameter vectors is taken from either (whichever is highest) a) all parameter vectors with maximum implausibility lower than 3 (which is the threshold suggested in Andrianakis et al., (2015)), or b) the 10% of remaining vectors with the lowest maximum implausibility. The ‘maximum implausibility’ is the maximum value of T(**x**) across all outcomes.

The process ended when any one of the following stopping criteria occurred:

- Less than 450 vectors remaining. *By definition, this would have found the small number of parameter vectors that have emulated values closest to the calibration data.*
- No outcomes with correlation greater than 0.6. *This would demonstrate that the signal from the training data cannot be detected above the noise of random variability, and hence it is not possible to develop more accurate emulators.*
- More than 90% of the remaining vectors have maximum implausibility lower than 3. *This would demonstrate that the emulators are not able to distinguish between plausible and implausible parameter vectors, and therefore drawing a sample from the remaining parameter vectors would be unlikely to produce training data that will in turn produce more accurate emulators.*

**References**

Andrianakis I, Vernon IR, McCreesh N, McKinley TJ, Oakley JE, Nsubuga RN, Goldstein M, White RG. Bayesian history matching of complex infectious disease models using emulation: a tutorial and a case study on HIV in Uganda. *PLoS Computational Biology,* 2015;11(1):e1003968.

Cobiac LJ, Scarborough P. Modelling future trajectories of obesity and body mass index in England. PLoS ONE 2016;11(12):e0167859.

Hankin RKS. Package ‘emulator’. CRAN. <https://cran.r-project.org/web/packages/emulator/index.html>

NatCen. Health Survey for England 2015 – trend tables. Available from: <https://digital.nhs.uk/data-and-information/publications/statistical/health-survey-for-england/health-survey-for-england-2015-trend-tables>

Oakley J. Estimating percentiles of uncertain computer code outputs. *Applied Statistics,* 2004;53(1):83-93.

Peters SAE, Huxley RR, Woodward M. Diabetes as risk factor for incident coronary heart disease in women compared with men: a systematic review and meta-analysis of 64 cohorts including 858,507 individuals and 28,203 coronary events. *Diabetologia,* 2014;57:1542-1551.

Prospective studies collaboration. Body-mass index and cause specific mortality in 900000 adults: collaborative analyses of 57 prospective studies. Lancet 2009; 373:1083-96.

Prospective Studies Collaboration. Age-specific relevance of usual blood pressure to vascular mortality: a meta-analysis of individual data for one million adults in 61 prospective studies. Lancer 2002; 360:1903-13.

Prospective studies collaboration. Blood cholesterol and vascular mortality by age, sex, and blood pressure: a meta-analysis of individual data from 61 prospective studies with 55000 vascular deaths. Lancet 2007; 370:1829-39.

Smolina K, Wright FL, Rayner M, Goldacre MJ. Determinants of the decline in mortality from acute myocardial infarction in England between 2002 and 2010: linked national database study. *BMJ,* 2012;344:d8059. For full data, see Oxford University DPhil thesis titled “Examination of the epidemiology of acute myocardial infarction in England using linked hospital and mortality data” by Kate Smolina, 2011.

Thun MJ, Apicella LF, Henley SJ. Smoking vs other risk factors as the cause of smoking-attributable deaths: confounding in the courtroom. JAMA. 2000 Aug 9;284(6):706-12.

**Figure S4.** MI incidence forecasts grouped by age groups


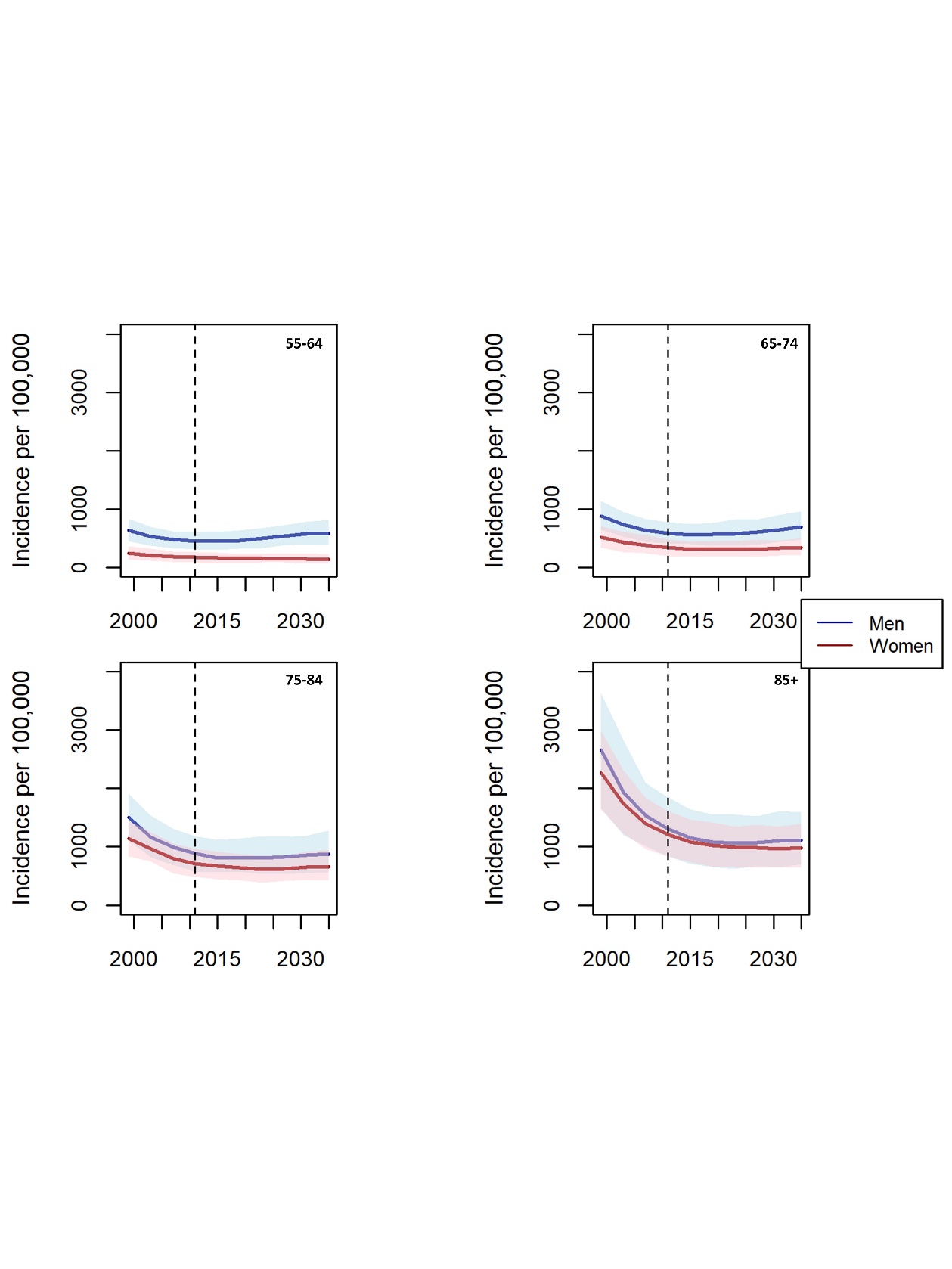


**Figure S5.** MI prevalence forecasts grouped by age groups


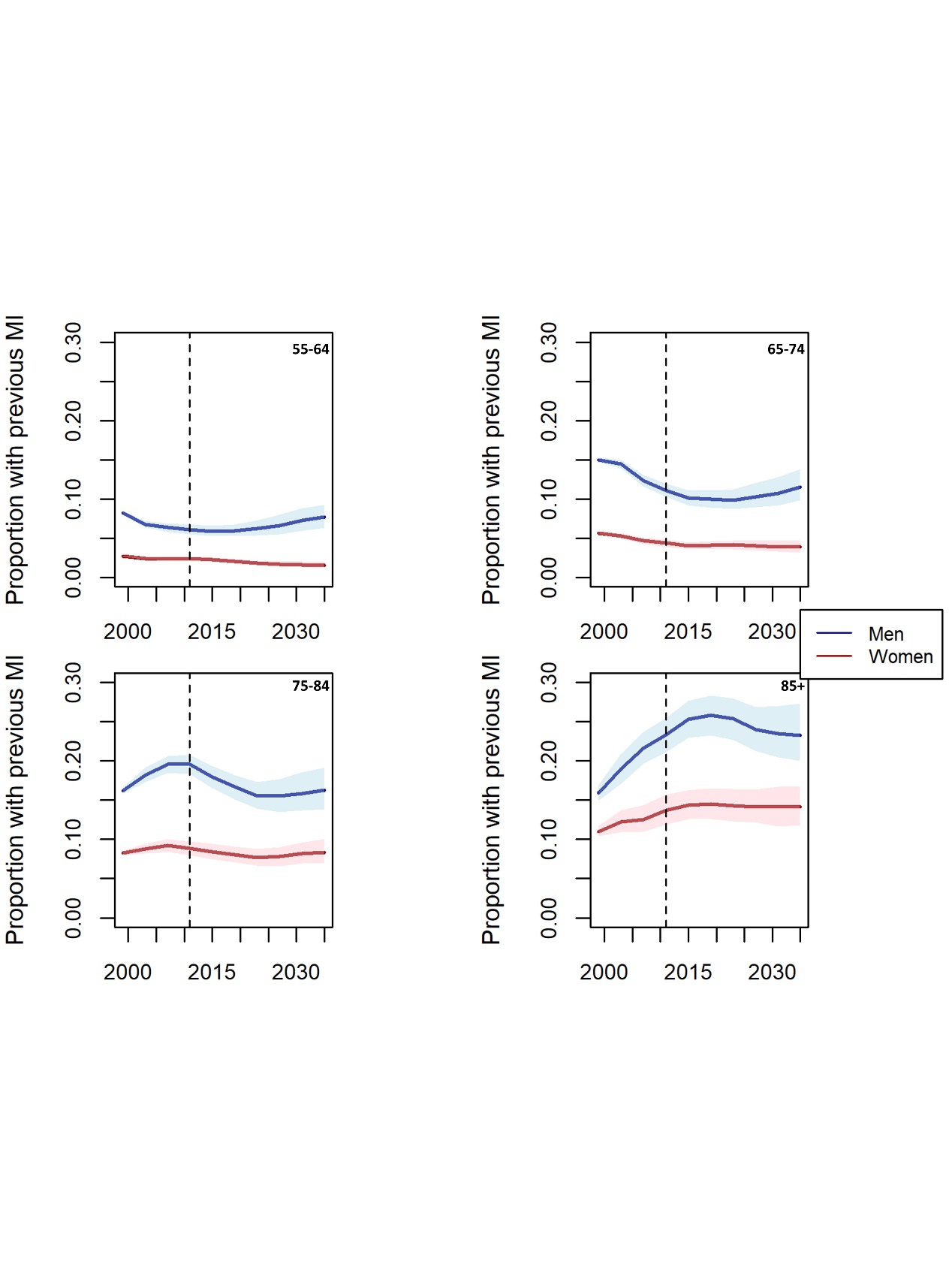

Supplement: S1 Text — (DOCX) [file pone.0270189.s001.docx]
